# Aorto-Uni-Iliac Stent Grafts with and without Crossover Femorofemoral Bypass for Treatment of Abdominal Aortic Aneurysms: A Parallel Observational Comparative Study

**DOI:** 10.1155/2015/962078

**Published:** 2015-12-03

**Authors:** Mohammed Elkassaby, Mahmoud Alawy, Mohamed Zaki Ali, Wael A. Tawfick, Sherif Sultan

**Affiliations:** ^1^Vascular Surgery Unit, Department of Surgery, Faculty of Medicine, Mansoura University, Mansoura, Egypt; ^2^Western Vascular Institute, National University of Ireland, Department of Vascular and Endovascular Surgery, University Hospital Galway, Newcastle Road, Galway, Ireland; ^3^The Galway Clinic, Royal College of Surgeon in Ireland, Dublin Road, Doughiska, Galway, Ireland

## Abstract

We investigated the safety and efficacy of primary aorto-uni-iliac (AUI) endovascular aortic repair (EVAR) without fem-fem crossover in patients with abdominal aortic aneurysm (AAA) and concomitant aortoiliac occlusive disease. 537 EVARs were implemented between 2002 and 2015 in University Hospital Galway, a tertiary referral center for aortic surgery and EVAR. We executed a parallel observational comparative study between 34 patients with AUI with femorofemoral crossover (group A) and six patients treated with AUI but without the crossover (group B). Group B patients presented with infrarenal AAAs with associated total occlusion of one iliac axis and high comorbidities. Technical success was 97% (*n* = 33) in group A and 85% (*n* = 5) in group B (*P* = 0.31). Primary and assisted clinical success at 24 months were 88% (*n* = 30) and 12% (*n* = 4), respectively, in group A, and 85% (*n* = 5) and 15% (*n* = 1), respectively, in group B (*P* = 0.125). Reintervention rate was 10% (*n* = 3) in group A and 0% in group B (*P* = 0.084). No incidence of postoperative critical lower limb ischemia or amputations occurred in the follow-up period. AUI without crossover bypass is a viable option in selected cases.

## 1. Introduction

Endovascular aortic aneurysm repair (EVAR) is currently an established safe and effective option for management of both elective abdominal aortic aneurysm (AAA) and ruptured abdominal aortic aneurysms (rAAAs).

There are many cases where endografting with bifurcated endoprosthesis is contraindicated due to anatomical restrictions, such as narrow terminal aorta and tortuous and narrow or calcified contralateral iliac artery [1, 2]. In these circumstances, aorto-uni-iliac endograft (AUI) and femorofemoral crossover bypass can overcome the limitations and exclude difficult AAAs [3, 4].

Recent studies confirm that AUI devices with occlusion of contralateral iliac artery and crossover femoral-femoral bypass have similar results as the treatment with bifurcated devices in selected patients [5]. This option is limited in daily practice for the treatment of AAA in fit patients. Nevertheless, it can be more convenient in cases of high-risk patients with complex iliac anatomy, r-AAAs, or redo surgery for failed grafts [5, 6].

The femorofemoral bypass step that comes with the standard use of AUI devices epitomizes an important disadvantage in this technique. This is due to the increased operative time and the added risk of the crossover, including graft infection, occlusion, wound complications, and peripheral vascular decompensation [7].

In our study, we report the feasibility of management of AAA in unfit patients, with AUI devices, without the need for femorofemoral bypass. This can be carried out in cases of previous chronic asymptomatic total occlusion of the contralateral iliac artery. We performed a parallel observational comparative study between the results of the standard practice and those with the omission of the fem-fem crossover.

## 2. Method

537 EVARs were implemented between January 2002 and July 2015 in University Hospital Galway, which is a tertiary referral center for aortic surgery and EVAR; of these 537 cases, 481 (89%) were on elective basis and 56 cases were emergency AAAs. The bifurcated configuration of the endografts represents 497 (92%) cases of the total number, while the aorto-uni-iliac configuration represents 40 (7.4%) cases, of which 34 cases where done with femorofemoral crossover bypass (12 emergencies) and six cases where done without the femorofemoral crossover bypass (3 emergencies). All aorto-uni-iliac devices were used primarily in these patients.

A prospective data record was kept for all patients. A retrospective analysis of the data was done to identify all patients with AUI devices implanted on an elective basis. Those who received a fem-fem crossover bypass were considered as the standard group (group A). Those treated with the omission of the bypass step were included in the study group (group B).

### 2.1. Patient Selection

Patient selection and measurements were based on preop CT angiography with a 3D reconstruction with the 3mensio Vascular software (3mensio Medical Imaging BV, Bilthoven, Netherlands). Ankle-brachial index (ABI) was measured in both sides of all patients preoperatively.

Total occlusion of one side of the iliac arteries (common or external iliac artery) was the prerequisite to consider the patient for omission of the crossover step. The decision then was based on strict criteria. These criteria included a minimum preop ABI of more than 0.5 and digital pressure more than 50 mmHg, with absent rest pain, tissue loss, or disabling claudication in the lower limb with occluded iliac axis. Patients in this group were all high-risk patients (ASA III+). Comorbid conditions and demographics of both groups are listed in Table 1.

Patients were kept under close observation and follow-up postoperatively. The contralateral limb vascularity was examined clinically using a hand held Doppler, observing capillary circulation in the foot, and monitoring any possible ischemic pain reported by the patient. This observation was carried out and recorded on hourly basis for the first 24 hours postoperatively. Any signs suggestive of development of an ischemic threat to the limb would be met by urgent readmission to the operative theatre for a completion crossover fem-fem bypass, but fortunately this did not take place for any of the study group patients.

### 2.2. Operative Procedure

All procedures were done under combined general and local anesthesia. Common femoral artery was the access vessel in all cases. Medtronic AUI stent grafts with proximal suprarenal bare stent were used in all cases, Talent up to 2008 and Endurant after this (Medtronic Inc., Minneapolis, MN, USA). The stent graft was deployed with 20% oversizing to aneurysm neck diameter.

**Figure 1:**
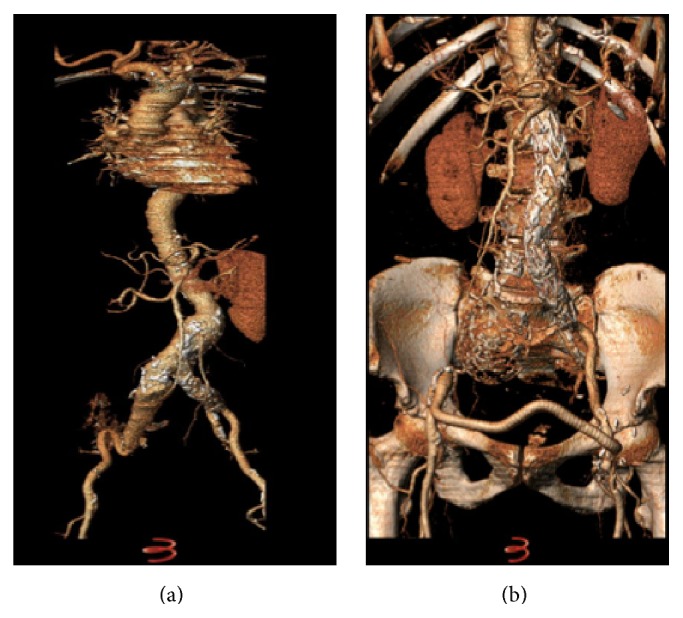
(a) 6 cm AAA with 7 cm right common iliac artery aneurysm. (b) AUI with left to right fem-fem and ligation of the distal right external iliac artery.

In group A, the contralateral iliac axis was occluded with an Endurant occluder (Medtronic Inc., Minneapolis, MN, USA) with 25% oversizing to the iliac artery diameter. The distal landing zone for the AUI graft was the common iliac artery in all cases, sparing the internal iliac artery on this side. The procedure was completed by a femorofemoral crossover bypass in the same setting, using a standard 8 mm diameter silver impregnated Dacron graft (Maquet Cardiovascular LLC, Wayne, NJ, USA) (Figure 1). We opted for the silver impregnated grafts to decrease the incidence of graft infection.

**Figure 2:**
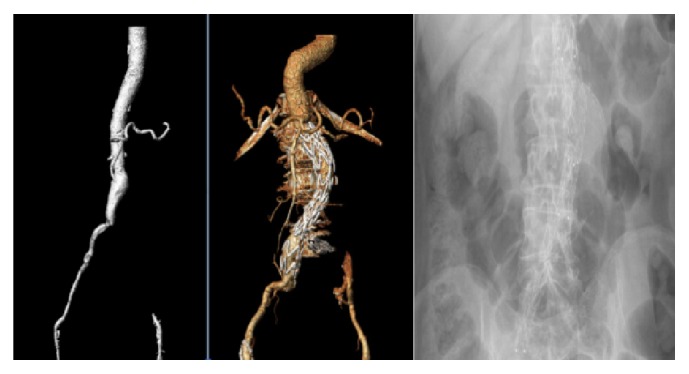
5.3 cm AAA in an 86-year-old female patient, with totally occluded left iliac system treated with an AUI EVAR without the need of either a left iliac occluder or fem-fem crossover graft.

In group B, the contralateral iliac artery was already occluded in all cases. The landing zone was the ipsilateral common iliac artery, sparing a patent internal iliac artery. This was performed with the aim of preserving the already present collaterals and to help the development of new collateral circulation to the other side (Figure 2).

### 2.3. Follow-Up

Follow-up reported is 24 months. Patients were followed up with abdominal colored duplex scan and ABI measurement in both lower limbs before discharge and in 6, 12, and 24 months' intervals. All patients gave informed consent for the procedure.

Statistical analysis was performed using the IBM SPSS 21 version (IBM SPSS Statistics for Windows, version 21.0, Armonk, NY: IBM Corp.). Comparisons were carried out using Mann Whitney* U* test and Fisher's exact test where suitable.

## 3. Results

The total number of patients included in the study is 40 patients: 34 in group A (standard) and 6 in group B (study). None of the patients were lost to follow-up in the first 24 postoperative months.

The mean age was significantly higher in group B compared to group A: 73.82 years compared to 69.58 years, respectively (*P* = 0.001).

The incidence of ischemic heart disease was significantly higher in group B compared to group A (67% to 18%, resp., *P* = 0.026). Only 14% of group A had chronic obstructive pulmonary disease (COPD), while 33% of group B had COPD (*P* = 0.045). All other comorbidities showed no statistically significant difference between the two groups. They are shown in Table 1 alongside with the demographics.

12 of group A cases (35%) were done on emergency basis including 3 ruptured AAAs, 6 cases symptomatic with abdominal or back pain, 2 cases of microembolization, and one case presenting with a small 3.1 cm AAA with trashing and acute thrombosis of both iliac arteries. This patient underwent urgent AUI endografting plus femorofemoral crossover to revascularize both lower limbs. ABIs were less than 0.5 in both side preoperatively in this patient, but they improved after surgery.

Three cases in group B (50%) were done as emergencies: 2 for ruptured AAA and 1 for abdominal and back pain.

The mean operative time was 114 minutes in group A and 79 minutes in group B (*P* = 0.013) and the mean blood loss was 346 mL in group A and was 220 mL in group B (*P* = 0.035). The mean fluoroscopy time was 10.2 minutes in group A compared to 9.5 minutes in group B (*P* = 0.488) and mean contrast loads were 76 mL and 72 mL in the same groups, respectively (*P* = 0.371); Table 2.

There was no perioperative mortality in either group. Primary and assisted technical successes were 97% (*n* = 33) and 3% (*n* = 1) in group A and were 85% (*n* = 5) and 15% (*n* = 1) in group B, respectively (*P* = 0.31). There was no perioperative mortality in either group. None of the aneurysms ruptured or were converted to open procedure during follow-up. Local groin wound hematoma occurred in 2 cases in group A. Both cases were managed with antibiotics and anti-inflammatory medications.

One case in each group had type II endoleak diagnosed after 6 months; both were kept under observation, and there was no increase in aneurysm sac diameter in both cases. This endoleak disappeared after 12 months of follow-up in the case from group A and after 24 months in the case from group B. One patient in group A exhibited type Ia endoleak without obvious graft migration. It was diagnosed after 6 months. This required realignment with aortic cuff, which was carried out successfully after 7 months from the initial procedure.

Distal graft migrations with type Ia endoleak was observed after 6 and 18 months in two cases in group A and were realigned using aortic cuffs within 1 month of their diagnosis. There was no difference in graft related complication between Talent or Endurant AUI configuration.

Major adverse clinical events (MACEs) observed were two cases in group A only. One of them was a case of moderate renal failure requiring temporary dialysis and the other was a case of myocardial infarction 1 day postoperatively, managed medically with no mortality. Graft infection, spinal cord ischemia, and distal embolization were not observed in any of the patients of both groups.

Primary and assisted clinical success at 24 months were 88% (*n* = 30) and 100% (*n* = 4), respectively, in group A, and they were 85% (*n* = 5) and 100% (*n* = 1), respectively, in group B (*P* = 0.125). Reintervention rate was 10% (*n* = 3) in group A and 0% in group B (*P* = 0.084). All postoperative results are illustrated in detail in Table 3.

Five patients in group A and two patients in group B had history of mild chronic ischemic claudication pain preoperatively. This was controlled with conservative medical treatment. None of the patients in group A or B reported any acute changes in their previous limb ischemic symptoms postoperatively.

**Figure 3:**
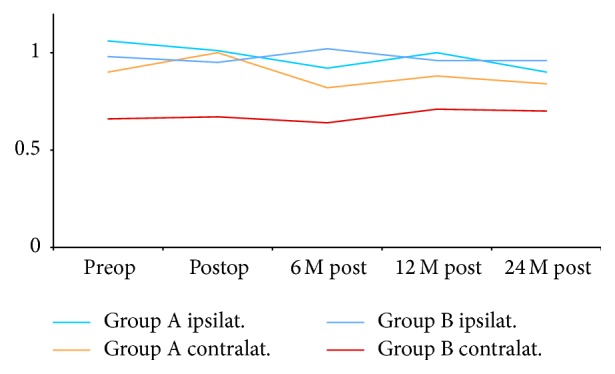
Mean ABIs follow-up in both lower limbs of patients of both groups. Group A is the standard practice of the AUI grafts with the fem-fem crossover. Group B is the study group with the omission of the bypass step.

ABI follow-up in both lower limbs of patients of both groups is illustrated in Figure 3. There was a statistically significant drop in the 24 months' postoperative mean ABI of the donor limbs in group A (*P* = 0.004), accompanied by a significant increase in that of the recipient limbs in the same group (*P* = 0.023). Changes in the 24 months' postoperative mean ABI of the AUI device limbs in group B were insignificant statistically (*P* = 0.203), while we observed a statistically significant increase in those of the occluded limbs in the same group (*P* = 0.005). This is shown in Table 4.

## 4. Discussion

AUI stent graft configuration was used early in the evolution of endovascular aneurysm repair because of its ability to load the stent graft through a smaller sheath and the ease of manufacture. Later on, this technique persisted even after development of other device configurations because of its ability to accommodate a greater number of aneurysms than the bifurcated design.

Placement of the AUI stent graft is technically less demanding than that of the standard bifurcated graft, which can be difficult and even impossible if anatomical constraints are severe. When placing an AUI, only one common iliac artery is stented. The absence of a contralateral limb ensures a smaller profile, which enables easier cannulation of smaller vessels. This also makes the device more pliable, enabling navigation of more difficult anatomy and making it possible to obtain a good seal in a less favorable aneurysms neck.

Another merit of this design is that no stent graft orientation is needed; and no contralateral limb cannulation is required. This allows less intra-aortic manipulations in 3-dimensional space under 2-dimensional fluoroscopic guidance and consecutively decreases the risk of embolization trashing the renal, visceral, or distal arteries. Also, a lesser amount of contrast agent is needed with this option, which minimizes the risk of contrast induced nephropathy.

To prevent back bleeding into the aneurysm sac, the contralateral common iliac artery is routinely occluded. Doing an extra-anatomical femorofemoral bypass ensures perfusion of the contralateral limb.

The simplicity of the AUI configuration makes it more suitable for high-risk patients and rAAA, as it requires less operative time and easier preoperative planning [3, 5]. On the other hand, one of the largest drawbacks to the use of AUI procedures is the reliance on extra-anatomic bypass grafts to revascularize the contralateral limb. These procedures are often complicated by the development of graft infection, graft occlusion, false aneurysm formation, and seromas in the groin.

Although many authors claim higher patency rates of the extra-anatomical femorofemoral bypass graft in cases of aneurysmal disease in comparison with occlusive disease [2, 8–10], they still admit the high rate of occlusion reported in cases of associated femoral and distal arterial disease [11, 12], which is the case in the patients reported in this study. Other local wound complications such as hematoma, seroma, false aneurysms, and superficial wound infection are also reported by many authors in relation to the use of synthetic graft for the femorofemoral bypass [8–10, 13]. In our study, in the study group of six patients (group B), all the patients had severe occlusive iliac artery disease; nevertheless, only two of them manifested with mild claudication pain preoperatively. This highlights the fact that iliac arterial occlusive disease is a common radiological finding in AAA patients even if this is not always manifested clinically to the same extent.

The fact that arterial occlusive disease was more obvious in the study group can explain the higher incidence of comorbidities in these patients as shown in Table 1. In our study, ischemic heart disease and chronic obstructive pulmonary disease were significantly higher in the study group compared to the standard group (*P* = 0.026 and *P* = 0.045, resp.). This could also predict higher incidence of complication according to a study [14] comparing the midterm results following the use of bifurcated and aorto-uni-iliac devices in the treatment of abdominal aortic aneurysms in 447 patients.

All patients in group B showed initial mild drop in ABI of both limbs. This came back to initial level in one-year time and even improved in the second year in four patients. This is shown in Figure 3.

Two of group A had localized wound hematoma and wound infection. They were treated with an aggressive course of antibiotics. Progression of the infection to the synthetic graft is a dreadful complication that might end in losing the limb or even worse. This situation might require a very invasive procedure with higher morbidity and mortality risk on the already fragile patient. Bonardelli et al. [15] reported the necessity for an ilioiliac bypass and removal of the infected femorofemoral bypass graft to salvage a patient with infected fem-fem bypass after AUI grafting for AAA.

Group A had 3 cases of type Ia endoleak; 2 of them were associated with distal graft migration and the 3rd one was without obvious migration. This is probably due to progression of the disease and dilatation of the neck. It can also be due to missed minor leak at the time of surgery with the 3rd case. We usually tend to stick to the upper limit of oversizing the graft to the neck diameter, especially if we are facing highly angulated or short neck which was the case with these patients.

Hynes and Sultan [16] reported that AUI EVAR with a fem-fem crossover is a safe and effective alternative as bifurcated endografts in high-risk patients with AAA. This is the same finding in our study. This is explained by the fact that AUI EVAR requires less preoperative planning, less operative time, and less trauma for those patients. In our study, the mean operative time was 114 minutes in the fem-fem group and 79 minutes in the no fem-fem group (*P* = 0.013). This short operative time is due to the omission of the bypass step. This also enabled less blood loss and less anesthesia time for those patients, which is more convenient when dealing with the high risk coming with severe comorbidities or ruptured AAAs. Jean-Baptiste et al. [14] reported higher incidence of complications with AUI EVAR compared to bifurcated endografts in high-risk patients. This could be attributed to the fact that AUI grafts were considered in this study for higher risk patients, while bifurcated grafts were used in more fit patients. Hynes and Sultan [16] published results in high-risk patients for bimodular devices which mirror the results of AUI with or without crossover but rebuff the finding of Jean-Baptiste that AUI EVAR is inferior to bimodular configuration. Carrafiello et al. [17] reported high mortality rate with AUI grafts, but a deeper look in this study design demonstrates that precious time was wasted in preoperative analysis and preoperative CT angiography in these unstable patients.

A number of studies [5, 13, 15, 18] suggested that the use of AUI instead of the bifurcated endografts might be attributed to its easier preoperative planning and intraoperative application, especially in centers with less experience with EVAR. This is not true in our study as more than 92% of our cases were performed by bimodular devices. Katsikas et al. [5] had documented through meta-analysis that the main advantages of the AUI endograft are its simplicity and versatility. In 2004, Arko et al. [1] demonstrated that fifty-five percent of patients considered for endovascular AAA repair met the anatomical selection criteria with men twice as likely as women. Conversely if we applied AUI options for those 220 patients, all can be managed routinely with this configuration. Currently up to 95% of our AAA patients are deemed suitable for EVAR. Clouse et al. [2] had proven that AUI with fem-fem crossover graft is a safe, effective option with satisfactory midterm results; however 5 of their patients had chronic contralateral iliac occlusion but they performed fem-fem crossover, in contrast to the 6 patients in our study that did not require crossover or any further intervention.

Our findings mirror those of Hinchliffe et al. [8] and Yilamaz et al. [9] that fem-fem crossover for AUI offers durable and encouraging long-term patency. Duplex follow-up is mandatory to detect early inadequate inflow or stenosis of the external iliac artery. Awareness of stent graft distortion or complications in the external iliac artery results in improved patency rates. The need for crossover must not discourage the use of AUI devices in patients with anatomy unfavorable bimodular devices as this simple step could be abandoned. Our results contradict the finding of Lipsitz et al. [10], as our results showed that crossover bypasses for aneurysmal disease are durable procedures either for pure aneurysmal disease or aneurysmal disease with aortoiliac occlusive pathology.

We understand that our study is limited by the small number of patients undergoing AUI EVAR. In our defense, this represents real life scenario highlighting the low percentage of use of AUI grafts in high volume centers for EVARs. AUI grafts represented only 7.5% of the total number of the performed EVAR procedures in our center. This is more or less similar to the percentage published in a recent study by Dortch et al. [19]. This may render our analysis prone to some bias. Another limitation is the lack of subgroup analysis comparing the study group to a group of patient with contralateral iliac occlusion who proceeded to have the crossover bypass. We had only one of those patients in the control group which rendered statistical comparison nonfeasible in this situation. We believe that, in spite of those limitation, the study still adds valuable information to the body of literature. These results can be reanalyzed in a future meta-analysis of similar studies.

## 5. Conclusion

AUI stent grafts are still a viable option for treatments of AAA, especially in cases of severe aortoiliac occlusive disease or comorbidities. Aortoiliac occlusive disease is a common radiological finding in AAA patients although it might not manifest clinically to the same degree. The femorofemoral crossover step routinely done with AUI configuration can be omitted in certain cases with asymptomatic total occlusion of one iliac axis. This can be done without compromising the vascularity of the contralateral limb. This furnishes us with the advantage of saving precious operative and anesthetic time in high-risk patients with severe comorbidities.

## Figures and Tables

**Table 1:** Demographics.

Demographics	Group A	Group B	*P* value
Patients	34	6	
Age (mean)	69.58	73.82	0.001
Gender M : F	3 : 1	1 : 1	0.319
Family history of AAA	6%	50%	0.018
Smoking history	70%	50%	0.169
Hyperlipidemia	85%	83%	0.216
Hypertension	65%	67%	0.195
Ischemic heart disease	18%	67%	0.026
Pulmonary Disease (COPD)	14%	33%	0.045
Diabetes	26%	0%	0.306
Chronic lower limb ischemia	14%	33%	0.095
Symptomatic AAA	35%	50%	0.082

**Table 2:** Operative details.

Mean	Group A	Group B	*P* value
Operative time (min)	114 (73–255)	79 (62–105)	0.013
Blood loss (mls)	346 (150–800)	220 (100–350)	0.035
Fluoroscopy time (min)	10.2 (6.8–19.7)	9.5 (7.5–16)	0.488
Contrast load (mls)	76 (57–118)	72 (48–92)	0.371

**Table 3:** Postoperative results.

24 months		Group A (*n* = 34)	Group B (*n* = 6)	*P* value
Technical success	1ry	33	5	0.311
	Assisted	1	1	
	2ry	0	0	

Clinical success	1ry	30	5	0.125
	Assisted	4	1	
	2ry	0	0	

Endoleak	Ia	3	0	0.046
	Ib	0	0	—
	Ic	0	0	—
	II	1	1	0.281

Limb salvage		100%	100%	—

MACEs (major adverse clinical events)		6%	0%	0.759

Reintervention rate		10%	0%	0.084

**Table 4:** Ankle-brachial index (ABI) follow-up.

		Preop mean ABI	24 months' mean ABI	*P* value
Group A	Donor limb	1.06	0.90	0.004
	Recipient limb	0.90	0.84	0.023

Group B	AUI limb	0.98	0.96	0.203
	Occluded limb	0.67	0.70	0.005
